# Mutation of the *Light-Induced Yellow Leaf 1* Gene, Which Encodes a Geranylgeranyl Reductase, Affects Chlorophyll Biosynthesis and Light Sensitivity in Rice

**DOI:** 10.1371/journal.pone.0075299

**Published:** 2013-09-10

**Authors:** Yong Zhou, Zhiyun Gong, Zefeng Yang, Yuan Yuan, Jinyan Zhu, Man Wang, Fuhai Yuan, Shujun Wu, Zhiqin Wang, Chuandeng Yi, Tinghua Xu, MyongChol Ryom, Minghong Gu, Guohua Liang

**Affiliations:** 1 Jiangsu Key Laboratory of Crop Genetics and Physiology/Key Laboratory of the Ministry of Education for Plant Functional Genomics, Yangzhou University, Yangzhou, Jiangsu, China; 2 Institute of Food Crops, Jiangsu Academy of Agricultural Sciences, Nanjing, Jiangsu, China; 3 Shanghai Academy of Agricultural Sciences, Shanghai, China; China Agricultural University, China

## Abstract

Chlorophylls (Chls) are crucial for capturing light energy for photosynthesis. Although several genes responsible for Chl biosynthesis were characterized in rice (*Oryza sativa*), the genetic properties of the hydrogenating enzyme involved in the final step of Chl synthesis remain unknown. In this study, we characterized a rice *light-induced yellow leaf 1-1* (*lyl1-1*) mutant that is hypersensitive to high-light and defective in the Chl synthesis. Light-shading experiment suggested that the yellowing of *lyl1-1* is light-induced. Map-based cloning of *LYL1* revealed that it encodes a geranylgeranyl reductase. The mutation of *LYL1* led to the majority of Chl molecules are conjugated with an unsaturated geranylgeraniol side chain. *LYL1* is the firstly defined gene involved in the reduction step from Chl-geranylgeranylated (Chl_GG_) and geranylgeranyl pyrophosphate (GGPP) to Chl-phytol (Chl_Phy_) and phytyl pyrophosphate (PPP) in rice. *LYL1* can be induced by light and suppressed by darkness which is consistent with its potential biological functions. Additionally, the *lyl1-1* mutant suffered from severe photooxidative damage and displayed a drastic reduction in the levels of α-tocopherol and photosynthetic proteins. We concluded that *LYL1* also plays an important role in response to high-light in rice.

## Introduction

Chlorophyll (Chls) molecules, which universally exist in photosynthetic organisms, play a central role in photosynthesis by harvesting light energy and converting it to chemical energy [1]. The Chl biosynthetic pathway was initially studied in Chl mutants of *Chlorella* [2]. Subsequently, Chls metabolism has been extensively analyzed in various organisms using biochemical and genetic approaches [3], [4], [5], [6]. Because the early enzymatic steps of Chl biosynthesis, from glutamyl tRNA to protoporphyrin IX, are shared with the heme biosynthetic pathway, many essential data regarding the identity of the associated enzymes were obtained from studies of non-photosynthetic organisms such as *Escherichia coli* [7]. The later steps of Chl biosynthesis are shared with the bacteriochlorophyll biosynthetic pathway [8], [9]. Directed mutational analysis using the photosynthetic bacterium *Rhodobacter capsulatus* had enabled the identification of genes involved in bacteriochlorophyll biosynthesis [5], and the homologous genes had been isolated from oxygenic plants [10]. To date, 27 genes encoding 15 enzymes in the chlorophyll biosynthetic pathway, from glutamyl-tRNA to Chl *a* and Chl *b*, have been identified in *Arabidopsis*, which represents angiosperm plants [11].

Chl consists of two moieties, Chlorophyllide (Chlide) and phytol, which are formed from the precursor molecules 5-aminolevulinate and isopentenyl diphosphate, respectively, in two different pathways, i.e., the tetrapyrrole and isoprenoid biosynthetic pathways. Both pathways provide the substrates, Chlide and geranylgeranyl pyrophosphate (GGPP), necessary for the final steps of Chl biosynthesis. The last step of Chl synthesis, after conversion of protochlorophyllide to Chlide, has been studied intensively since geranylgeraniol was first identified as the esterifying alcohol of protochlorophyll a in pumpkin seeds [12]. Soll et al. suggested that there are two pathways for Chl biosynthesis [13]. In one pathway, GGPP synthesized in the chloroplast stroma is esterified to Chlide by Chl synthase in the thylakoid membranes, and the product Chl-geranylgeranylated (Chl_GG_) is reduced stepwisely via Chl-dihydrogeranylgeraniol (Chl_DHGG_) and Chl-tetrahydrogeranylgeraniol (Chl_THGG_) to Chl-phytol (Chl_Phy_) [13], [14], [15]. Recombinant Chl synthase, encoded by the *G4* gene of *Arabidopsis* [16] and overexpressed in *Escherichia coli*, also esterifies Chlide preferentially with GGPP to form Chl_GG_ [17]. The bchP gene product of *Rhodobacter sphaeroides* is required for the three steps of the isoprenoid moiety of bacteriochlorophyll necessary for the reduction of Chl_GG_ to Chl_Phy_ [6], [18], [19]. In the other pathway, GGPP is reduced in the envelope membranes to phytyl pyrophosphate (PPP), which is then transferred to the thylakoid membranes, where Chl synthase directly generates Chl_Phy_ [18], [19]. Chl synthase derived from the *ChlG* gene of *Synechocystis* and bacteriochlorophyll synthase encoded by the *Rhodobacter bchG* gene give preference to PPP relative to GGPP [20].

The three-step hydrogenation of GGPP into PPP and Chl_GG_ into Chl_Phy_ is catalyzed by NADPH-dependent geranylgeranyl reductase [5], [13], [17]. Reduced activity of geranylgeranyl reductase leads to the loss of Chl_Phy_ and the accumulation of Chl_GG_, Chl_DHGG_ and Chl_THGG_. Geranylgeranyl reductase overexpressed in *Escherichia coli* catalyzes the stepwise hydrogenation of Chl_GG_ to Chl_Phy_. Several genes encoding geranylgeranyl reductase were characterized in prokaryotes [5], [21], [22], [23], [24] and higher plants such as *Arabidopsis* [17], tobacco [15], peach [25] and olive [26].

In this study, we characterized a rice mutant *lyl1-1* (*light-induced yellow 1eaf 1-1*) from *japonica* c.v. Zhonghua 11 (ZH11), displaying a dynamic yellow-green leaf phenotype, reduced level of Chl, arrested development of chloroplasts and hypersensitive to light. Map-based cloning of *LYL1* revealed that this gene encodes a geranylgeranyl reductase. A single nucleotide C-to-T substitution in the coding region resulting in an amino acid change from an alanine residue to valine was found in the *lyl1-1* mutant. We provided evidence that *LYL1* simultaneously participates in the synthesis of Chl_Phy_ and α-tocopherol in rice.

## Results

### Characterization of a chlorophyll-deficient rice mutant

**Figure 1:**
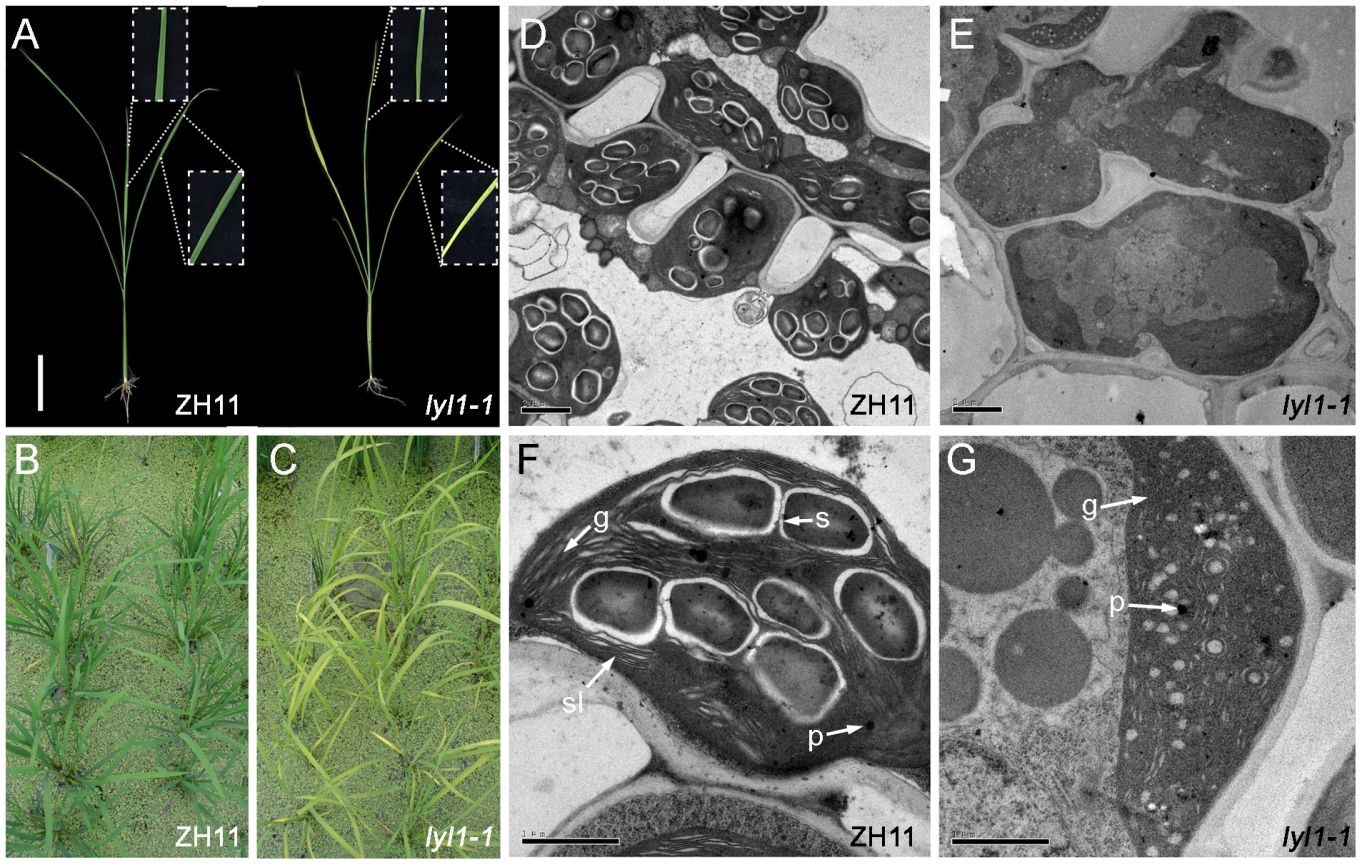
Phenotype of the rice *lyl1-1* mutant. (A) wild type ZH11 (left) and *lyl1-1* mutant (right) at the seeding stage. (B) ZH11 plants at tillering stage. (C) The *lyl1-1* plants at tillering stage. (D–G), The electron microscopic analysis of ZH11 and *lyl1-1* leaves. (D) and (E), The mesophyll cells of ZH11 and *lyl1-1* mutant. Bar  = 1 µm. (F) and (G), The chloroplasts of ZH11 and *lyl1-1* mutant. g, grana stack; p, plastoglobule; s, starch granule; sl, stroma lamellae. Bar  = 2 µm.

To investigate the molecular nature of rice chlorophyll metabolism, a light-induced yellow leaf mutant, designated as *lyl1-1*, was isolated from the progeny of a *japonica* rice ZH11 treated with ^60^Co. Phenotypic observation showed that the *lyl1-1* mutant grew slowly and produced premature yellowing leaves under natural conditions. The young leaves from leaf sheaths stayed green without any visible chlorosis (Figure 1 A–C). However, the leaves rapidly turned yellow in several days. To characterize the yellow leaf phenotype of *lyl1-1*, we measured the Chl content. The contents of Chl *a*, Chl *b* and total Chl in the *lyl1-1* mutant were 25.8% to 40.6%, 33.0% to 41.0%, and 30.8% to 40.4% of these in ZH11 plants, respectively, in different growth stages (Table 1). These results indicated that the yellow leaves of the *lyl1-1* mutant resulted from reduced Chl levels.

**Table 1:** Chl content in leaves of wild type ZH11 and *lyl1-1*, in mg/g fresh weight.

Growth Stage	Line	Total Chls	Chl *a*	Chl *b*	Chl *a*/Chl *b*
Seeding stage	ZH11	3.07±0.11	2.24±0.07	0.83±0.04	2.70±0.15
	*lyl1-1*	1.24±0.01	0.91±0.01	0.34±0.01	2.68±0.06
Tillering stage	ZH11	3.77±0.23	2.91±0.01	1.09±0.01	2.67±0.08
	*lyl1-1*	1.16±0.08	0.75±0.02	0.36±0.02	2.08±0.11
Heading stage	ZH11	3.95±0.02	3.00±0.05	0.95±0.06	3.15±0.26
	*lyl1-1*	1.31±0.06	1.00±0.05	0.31±0.00	3.23±0.13

Chls were measured in 95% ethanol extracts from the first, second and third leaves from top at the indicated growth stages. Values shown are the mean ±SD.

We further investigated the ultrastructure of chloroplasts using transmission electron microscopy. In ZH11 plants, the chloroplasts displayed well-developed membrane systems composed of grana connected by stroma lamellae (Figure 1D, F). Grana stacks in the *lyl1-1* mutant, however, appeared less dense and lacked grana membranes compared to those in ZH11. The thylakoid membrane systems of chloroplasts were disturbed in the *lyl1-1* mutant, and the membrane spacing was not as clear as that in ZH11 chloroplasts (Figure 1E, G). Therefore, the development of chloroplast thylakoid was suppressed in the *lyl1-1* mutant.

**Figure 2:**
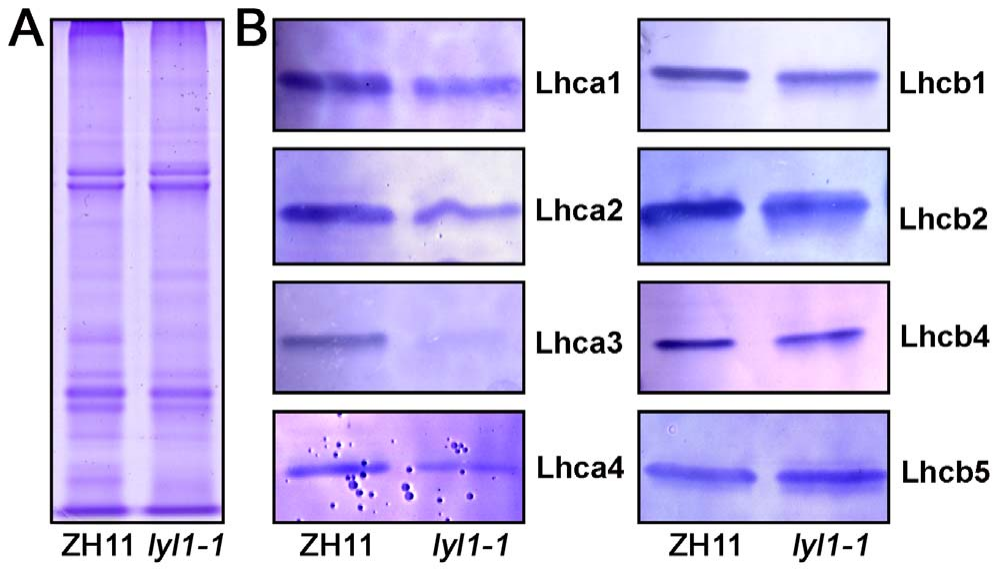
Gel blot analysis of proteins from ZH11 and *lyl1-1* leaves. (A) A coomassie blue-stained gel of thylakoid membrane proteins was provided to show equal protein loading. (B) Gelblotting of the LHC proteins. Proteins were extracted from the first, second and third leaves from top of plants at tillering stage. Each lane was loaded with 20 mg of extract.

We performed gel blot analysis to examine the abundance of LHC proteins (Light-harvesting chlorophyll-binding proteins) (Figure 2). All LHCI proteins examined were found to be poorly accumulated in the *lyl1-1* mutant. Lhca 3 was almost undetectable. Two major trimeric LHCII proteins, Lhcb1 and Lhcb2, and one monomeric LHCII protein, Lhcb4, were also inhibited in the *lyl1-1* mutant. However, the accumulation of Lhcb5 was not affected.

### The yellowing of *lyl1-1* mutant was caused by high-light stress

**Figure 3:**
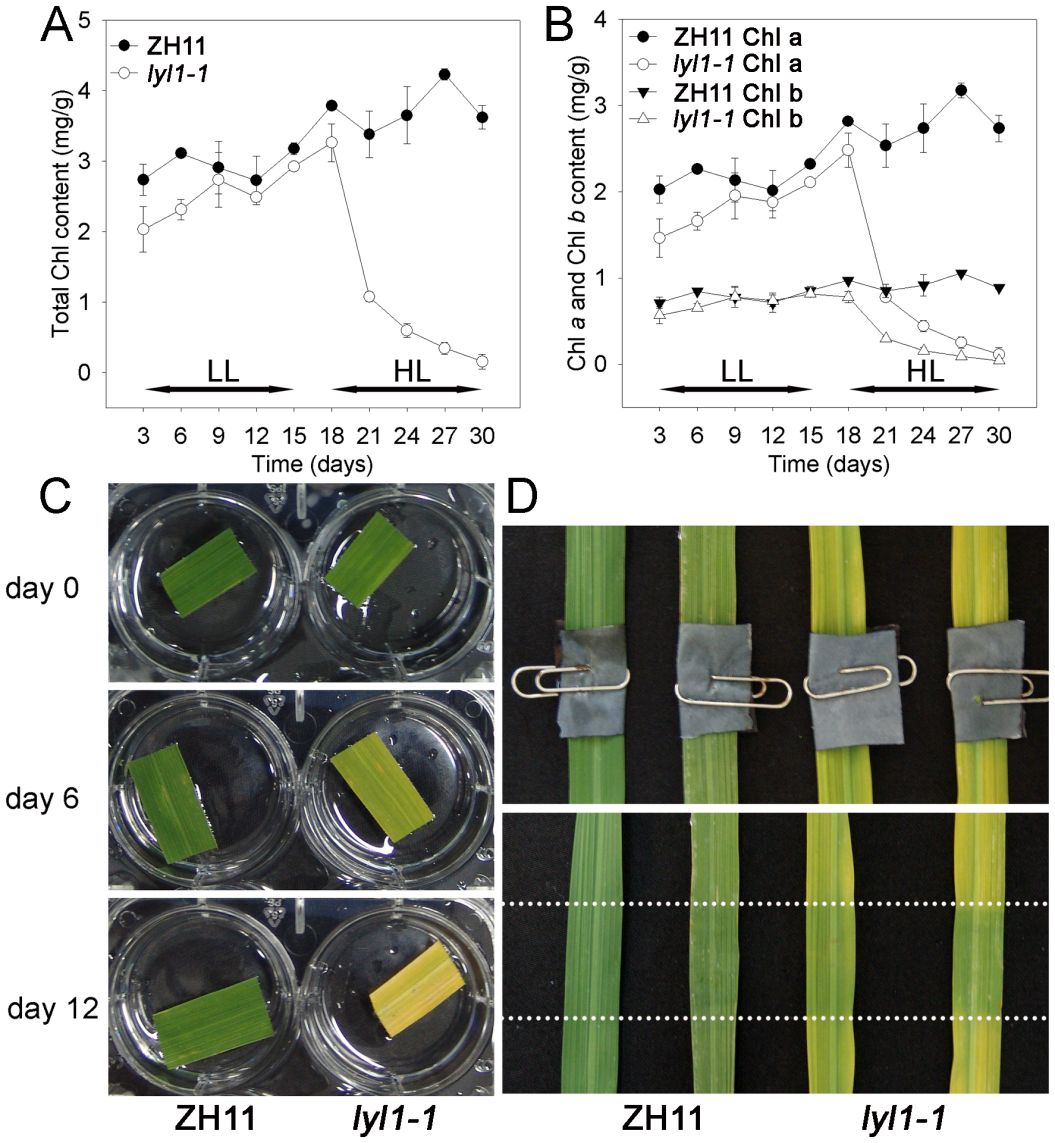
Effects of light intensity on leaf Chl content of ZH11 and *lyl1-1*. (A) Comparison of the total Chl content of ZH11 and *lyl1-1* plants exposed to different light intensities. (B) Comparison of the Chl *a* and Chl *b* contents of ZH11 and *lyl1-1* plants exposed to different light intensities. (C) Phenotype of ZH11 and *lyl1-1* leaves grown under high-light conditions for various periods of time (0, 6 and 12 days). Plants grown under low-light (LL, 100 µmol photonm m^−2^ s^−1^) at a 12 h photoperiod at 27°C for 30 days were transferred to high-light conditions (HL, 400 µmol photon m^−2^ s^−1^) at 27°C. The leaves were detached from the illuminated plants and photographed in water. (D) Light-shading experiment. Plants were initially grown under low-light conditions. A black integument covered in the leaf center to block out light before transfer to high-light conditions (up). The same leaves are shown on day 6 after high-light stress (down). The parts between the two dotted lines were covered by black integuments.

To reveal whether the green-yellow transformation of *lyl1-1* leaves depend or independent on environmental factors, we tested the response of *lyl1-1* to different light and temperature treatments. Plants were first grown under low-light conditions (100 µmol photon m^−2^ s^−1^) and subsequently transferred to high-light conditions (400 µmol photon m^−2^ s^−1^) at 27°C. As shown in Figure 3, under low-light conditions, the *lyl1-1* mutant displayed a phenotype similar to that of ZH11. The content of total Chl in *lyl1-1* was slightly lower than that in ZH11 (Figure 3A). After the transition to high-light conditions, the total Chl content in *lyl1-1* rapidly decreased from 2.93 mg/g to 0.16 mg/g, whereas the Chl content in ZH11 increased from 3.18 mg/g to 3.62 mg/g (Figure 3A, C). In addition, the changes in Chl *a* level occurred at a similar rate to that of Chl *b* in ZH11 and *lyl1-1* (Figure 3B). Experiments in which high-light was replaced by various temperatures (data not shown) indicated that temperature was not responsible for the observed yellowing. Taken together, the yellowing and light hypersensitivity of the *lyl1-1* mutant may be caused by high-light stress.

To confirm this conclusion, we carried out a shading experiment. Plants were initially grown under low-light conditions. A black integument covered the center part of leaves to block out light before the plants were transferred to high-light conditions. After 6 days of exposure, the part of *lyl1-1* leaf shaded by the integument remained green, but the remaining leaf turned yellow. No significant difference was observed in ZH11 leaf exposed to same conditions (Figure 3D). This observation directly suggested that the yellowing of *lyl1-1* is light-induced and the mutation of *LYL1* gene enhances the photosensitivity of rice leaves.

### Map-based cloning of *LYL1*

For genetic analysis of the *lyl1-1* mutant, an F_2_ population was constructed from the cross between *lyl1-1* and 9311. All of the F_1_ plants displayed a normal green leaf phenotype. The normal leaf and yellow leaf plants of the F_2_ population showed a segregation ratio of 3∶1 (*X* ^2^ = 0.66<*X* ^2^_0.05,1_ = 3.84), which suggests that the yellow leaf phenotype in the *lyl1-1* mutant is controlled by a single recessive nuclear gene.

**Figure 4:**
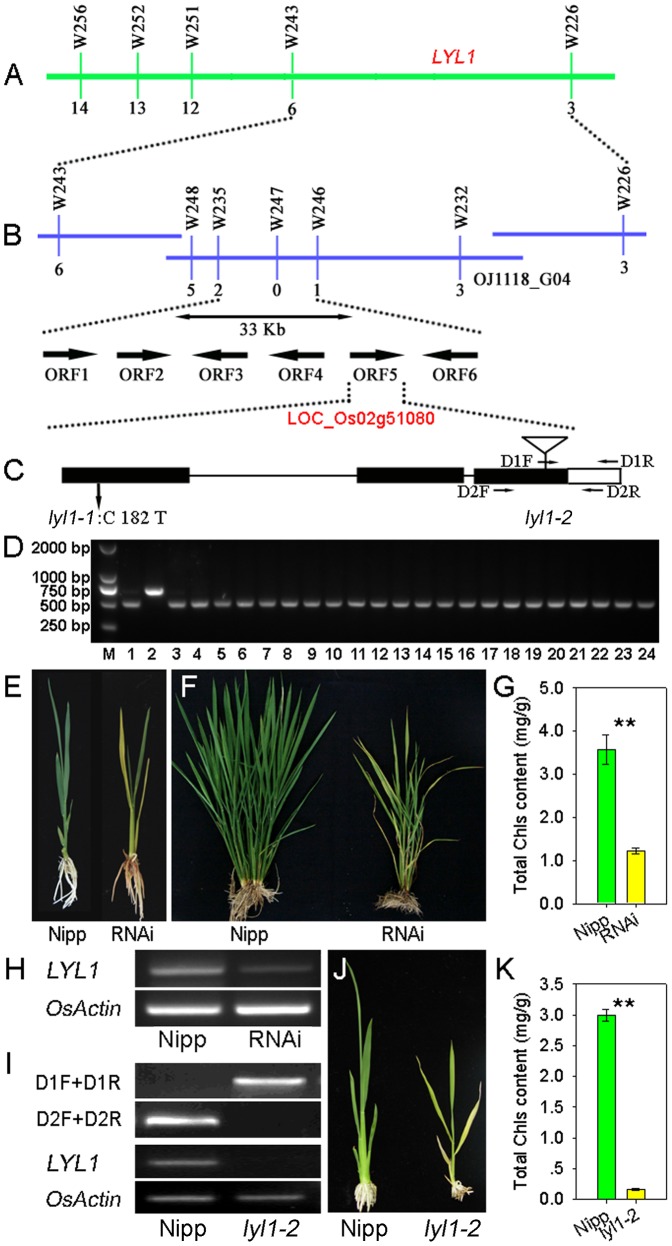
Map-based cloning of *LYL1*. (A) Rough mapping of the *LYL1* locus. *LYL1* is located between two markers, W243 and W226, on the long arm of chromosome 2. (B) Fine mapping of the *LYL1* locus. The *LYL1* gene is limited in a 33-kb genomic DNA region between markers W235 and W246, and it co-segregates with marker W247. Six candidate genes are located within this region in the Nipponbare genome, according to the TIGR Rice Genome Annotation Database. LOC_Os02g51080 is the candidate for *LYL1*. (C) *LYL1* gene structure at the genomic level. Three exons and the mutation positions are indicated. (D) Confirmation of the splice variation by analysis of the PCR amplicon size in the *lyl1-1* mutant and 22 rice cultivars. Lane 1, ZH11; lane 2, *lyl1-1*; lane 3-24, normal rice varieties, Zhongxian 3037, Tianegu, Wuxiangjing 3, Wuyunjing 8, Wuxiangjing 9, 9915, Nantehao, Wu 2661, Nanjing 46, Guandong 194, Nippobare, Guangluai 4, Balillar, Wuyunjing 7, 9516, 3015, Dular, 9311, Wujing 5, Miyang 23, Zhengdao 88 and Fengsizhan. (E) Phenotype of a 3-week-old RNAi transgenic plant. (F) Phenotype of a 2-month-old RNAi plant. (G) Comparison of Chl content between wild type Nipponbare and RNAi line. (H) Examination of *LYL1* expression level in the RNAi line by RT-PCR. *OsActin* was amplified as a control. (I) PCR and RT-PCR identification of the Tos17 insertion mutant *lyl1-2*. D1F was a primer derived from the Tos17 region. The D1R, D2F and D2R primers were derived from the genes examined. For RT-PCR analysis, *OsActin* was amplified as a control. (J) Phenotype of a 3-week-old *lyl1-2* mutant. (K) Comparison of Chl content between Nipp and *lyl1-2*. Data presented are mean ±SD. ** Significant at the 0.01 level.

The *LYL1* gene was initially mapped between the markers W243 and W226 on the long arm of chromosome 2 (Figure 4A), using the F_2_ population derived from *lyl1-1* and 9311. A comparison of chromosomal locations and leaf phenotypes indicated that *LYL1* is a novel gene and different from previously identified genes related to leaf color alteration. For fine mapping of *LYL1*, more than 6000 F_2_ individuals were developed and new InDel markers between W243 and W226 were designed according to sequence differences between *indica* and *japonica* rice (Table S2). Five markers exhibiting polymorphisms between the *lyl1-1* mutant and 9311 were used to screen recombinants. Using 1203 recessive plants, the *LYL1* gene was subsequently limited to a 33-kb region between the markers W246 and W232 on a single BAC clone, OJ1118_G04 (Figure 4B). Within this DNA segment, six open reading frames (ORFs) have been predicted according to the Rice Genome Annotation Project (http://rice.plantbiology.msu.edu/cgi-bin/gbrowse/rice/). All genes within this region were amplified and sequenced. A single nucleotide C-to-T substitution at position 182 in the coding region was found in the first exon of LOC_Os02g51080 in *lyl1-1*. This substitution results in a change from an alanine residue to valine (Figure 4C). No other DNA sequence change was detected in other candidates. To examine whether the C-to-T mutation was present as a natural variant in other cultivars, we performed CAPS analysis of 22 typical *indica* and *japonica* rice cultivars, as the SNP in *lyl1-1* removed the original *Sac*II site. And all 22 cultivars exhibited the original restriction fragment (Figure 4D). Thus, LOC_Os02g51080 is a good candidate gene for *LYL1*.

To confirm that the SNP mutation in LOC_Os02g51080 is responsible for *lyl1-1*, we utilized an RNA interference (RNAi) approach to knockdown this gene. Eleven transgenic plants expressing an inverted repeat of LOC_Os02g51080 were generated in Nipponbare (Nipp). Among these, nine plants displayed the Chl deficient phenotype (Figure 4E–H). In addition, a TOS17 retrotransposon insertion mutant, *lyl1-2*, was identified (line number, NE1041; Figure. 4C, I). The Tos17 insertion located in the exon 3 of LOC_Os02g51080 and no transcript of LOC_Os02g51080 can be detected in the *lyl1-2* mutant (Figure 4I). As expected, the *lyl1-2* mutant exhibited yellow leaves with significantly reduced Chl level (Figure 4J, K). Therefore, the yellow leaves phenotype of *lyl1* mutant was indeed caused by an SNP mutation in LOC_Os02g51080.

### *LYL1* encodes a geranylgeranyl reductase with FAD binding domain

Sequence comparison between genomic DNA and cDNA revealed that the *LYL1* gene comprises three exons and two introns and encodes a 463-amino acid protein with a molecular mass of approximately 50 kDa. The C-to-T substitution resulted in a change from an alanine residue to valine in the encoded protein in *lyl1-1*. A protein BLAST search showed that *LYL1* encodes a geranylgeranyl reductase with an FAD binding domain. One homolog having 57% sequence identity with *LYL1*, named as *LIL2* (LOC_Os01g16020), was found in rice genome.

**Figure 5:**
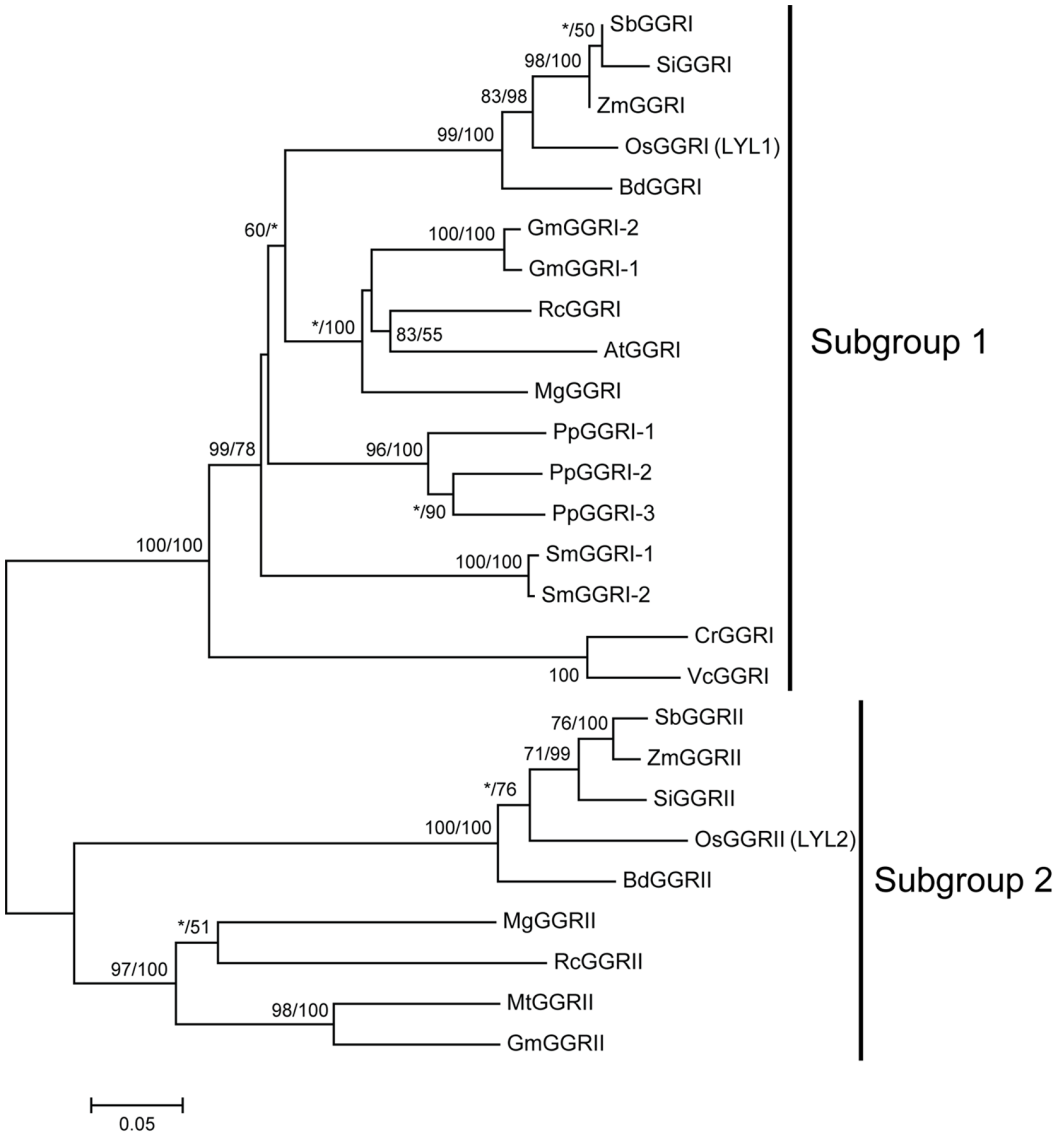
Phylogenetic tree of green plant *LYL1* proteins. The numbers above the branches show bootstrap values for maximum likelihood and distance analysis, respectively. Asterisks indicate values lower than 50%. The locus name for each gene was from the database of Phyzotome (v9.0). A species acronym is included before the gene name: At, *Arabidopsis thaliana*; Bd, *Brachypodium distachyon*; Cr, *Chlamydomonas reinhardtii*; Gm, *Glycine max*; Mt, *Medicago truncatula*; Mg, *Mimulus guttatus*; Os, *Oryza sativa*; Pp, *Physcomitrella patens*; RC, *Ricinus communis*; Sb, *Sorghum bicolor*; Si, *Setaria italica*; Sm, *Selaginella moellendorffii*; Vc, *Volvox carteri*; Zm, *Zea mays*.

To illustrate the domain structure of LYL1 protein, we searched the Pfam database and found that it only contained a pyridine nucleotide-disulphide oxidoreductase domain (CL0063), a signature of the FAD super family. Blast searches also revealed that genes encoding FAD binding proteins exist widely in green plants, unicellular green algae, mosses, lycophytes and angiosperms. Although a whole genome sequence has not yet been found in gymnosperms, several ESTs from *Picea sitchensis*, *P. glauca*, *Pinus taeda* and *P. contorta* showed high similarity with *LYL1* gene. To explore the phylogenetic relationship of these genes, we characterized homologs from the species representing the main lineages of green plants, including the green algae *Chlamydomonas reinhardtii* and *Volvox carteri*, the moss *Physcomitrella patens*, the lycophyte *Selaginella moellendorffii* and five monocot and five dicot angiosperms (Table S1). The amino acid length of these selected plant proteins ranged from 442 to 524. The FAD binding domain genes of green plants were placed into two groups with 100% bootstrap values in both the ML- and NJ-generated phylogenetic trees (Figure 5). On the phylogenetic tree, all monocot species that were tested contain genes in both groups, while some dicot species seems to have lost genes. For example, *Arabidopsis* appears to have lost the gene from subgroup 2, while *Medicago* lost the group 1 gene. Furthermore, all the genes from non-seed plants (including green algae, moss and lycophyte) were assigned to group 1, illustrating that subgroup 2 was formed independently in seed plants.

We searched the *nr* and EST information in NCBI databases, as well as available eukaryotic genome databases, and found that homologs of green plant geranylgeranyl reductase genes exist in bacteria and algae, including red algae, brown algae, diatoms and others. We selected representative homologs from each taxonomical group of cellular organisms to build a large phylogenetic tree (Figure S1, S2). The existence of homologous genes in multiple plants, bacteria and algae suggests that there is a widely conserved mechanism for the transformation from Chl_GG_ to Chl_phy_ and GGPP to PPP across divergent species.

### Expression patterns of *LYL1*

**Figure 6:**
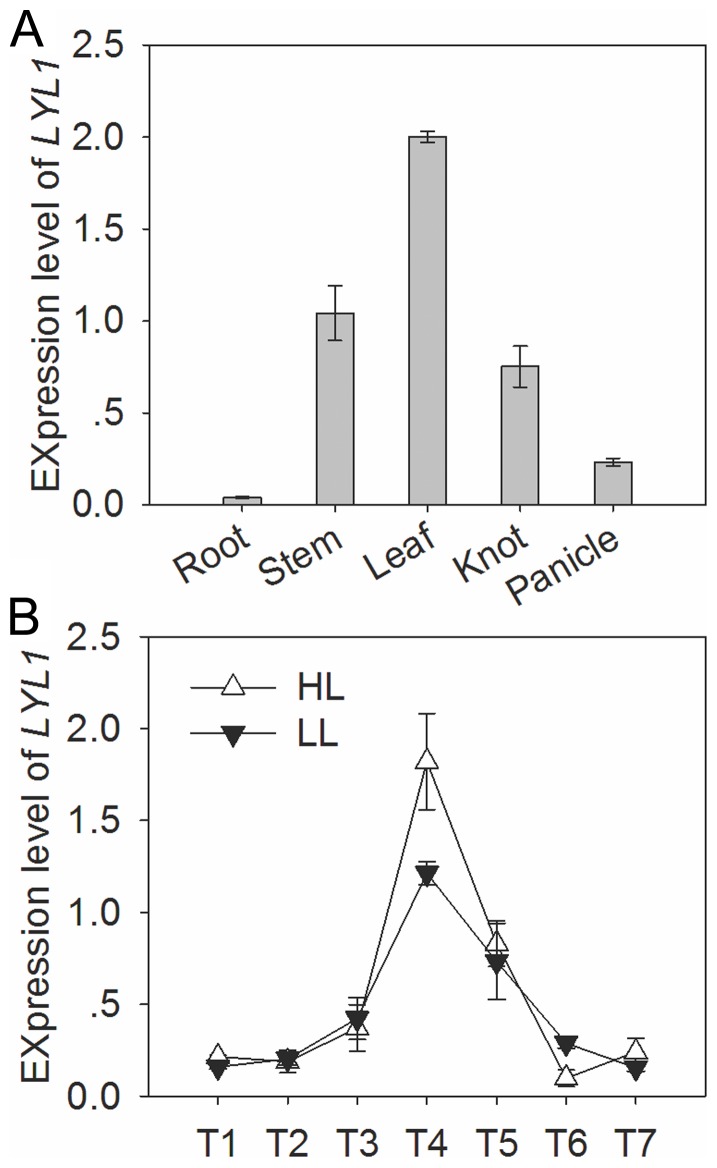
Expression analysis of rice *LYL1* gene. (A) Transcript levels of *LYL1* relative to *OsActin* in various tissues detected by quantitative real-time PCR. (B) Time-course of *LYL1* expression in response to light and dark conditions. About 3-week-old seedlings under dark, low-light (LL, 100 µmol photonm m^−2^ s^−1^) and high-light conditions (HL, 400 µmol photon m^−2^ s^−1^) at 27°C were used for expression analysis. T1, 12 hours of dark treatment; T2–T4, 3, 6 and 9 hours after the initiation of light treatment; T5–T8, 3, 6 and 9 hours after dark exposure.

Quantitative real-time PCR analysis showed that *LYL1* is constitutively expressed in organs such as root, stem, leaf, knot and panicle (Figure 6A). However, the expression in leaf was relatively high, while and the expression in root was almost non-existent or at a very low level, indicating that the *LYL1* gene has a specific expression pattern.

We examined the effects of light and dark growing conditions on the expression of *LYL1*, and found an obvious time-course change in expression levels was observed when plants were grown under light or dark conditions. The *LYL1* transcript level was relatively low during the dark period, but rose rapidly after 6 h under low-light condition (400 µmol photon m^−2^ s^−1^) and achieved a 9-fold increase during 9 h. *LYL1* expression decreased upon exposure to darkness and was restored within 6 h (Figure 6C). To test whether *LYL1* is differentially expressed under low-light conditions, we further analyzed the transcript accumulation in leaves exposed to a decreasing light intensity to 100 µmol photon m^−2^ s^−1^. This exposure can also induce the expression level and resulted in a 6-fold increase of *LYL1* transcripts at the maximal level. These results clearly demonstrated that *LYL1* is an expressed light-responsive gene, which is consistent with its potential biological functions. Actually, most of rice genes involved in the chlorophyll biosynthesis pathway are light-responsive [27], [28].

### Mutation of *LYL1* leads to an accumulation of Chl intermediates and a deficiency of α-tocopherol

**Figure 7:**
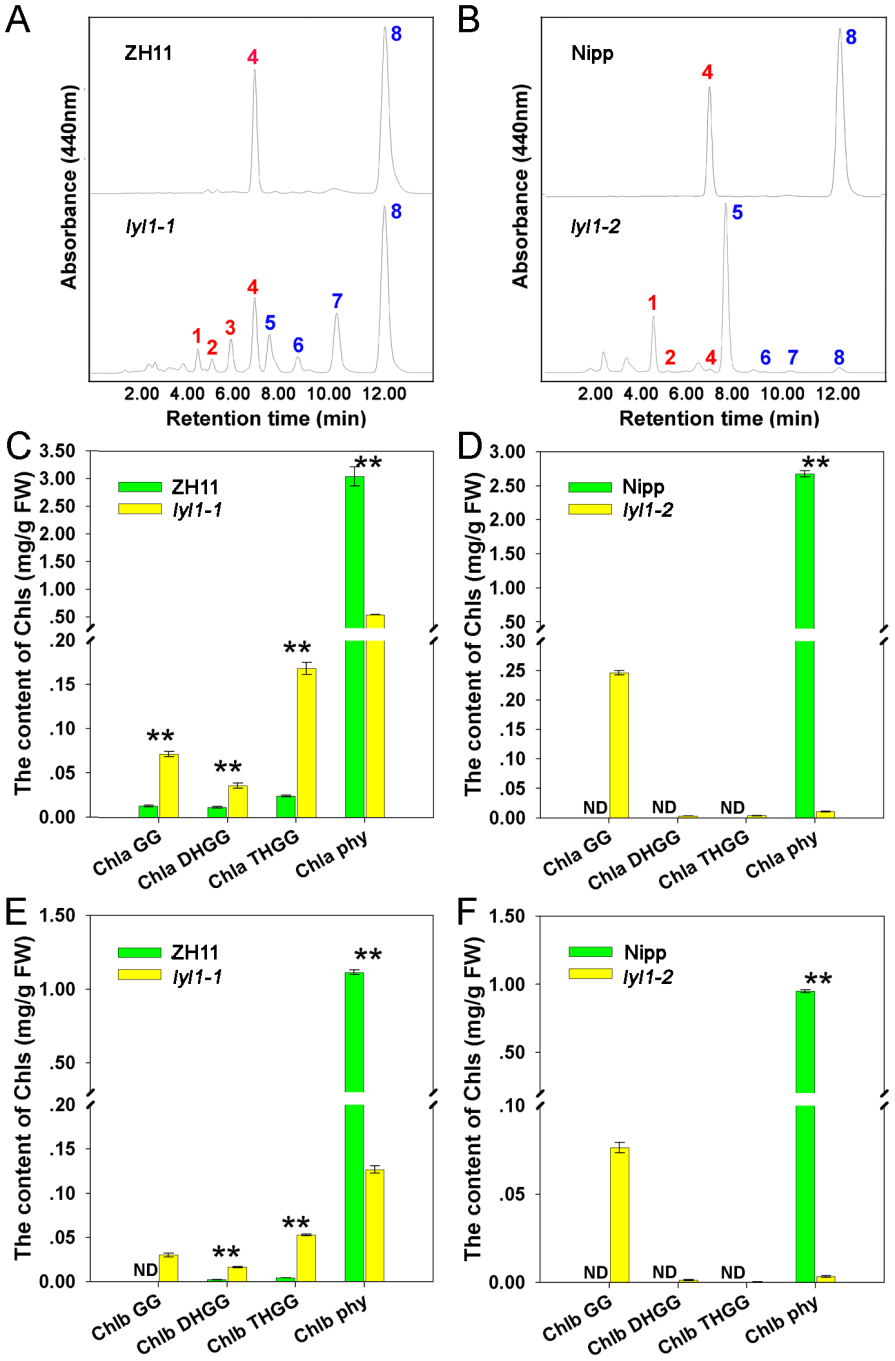
HPLC analysis of the accumulation of Chl derivatives. The fluorescence intensity at 650(A) HPLC chromatograms of extracts from leaves of ZH11 (top) and *lyl1-1* (bottom). (B) HPLC chromatograms of extracts from leaves of Nipp (top) and *lyl1-2* (bottom). Peak 1, Chl *b*_GG_; peak 2, Chl *b*_DHGG_; peak 3, Chl *b*_THGG_; peak 4, Chl *b*_phy_; peak 5, Chl *a*_GG_; peak 6, Chl *a*_DHGG_; peak 7, Chl *a*_THGG_; and peak 8, Chl *a*_phy_. (C) The contents of Chl *a* derivatives in ZH11 and *lyl1-1*. (D) The contents of Chl *a* derivatives in Nipp and *lyl1-2*. (E) The contents of Chl *b* derivatives in ZH11 and *lyl1-1*. (F) The contents of Chl *b* derivatives in Nipp and *lyl1-2*. The extracts were isolated from the first, second and third leaves of ZH11, *lyl1-1*, Nipp and *lyl1-2* plants grown under natural conditions (high light). Data presented are mean ±SD. ND =  Not Detected. The Chl *b*_GG_ of ZH11 and Chl intermediates in Nipp were not detected. ** Significant at the 0.01 level.

Geranylgeranyl reductase is an NADPH-dependent enzyme and responsible for the reduction of GGPP to PPP and the reduction of Chl_GG_ to Chl_phy_. To identify the function of *LYL1* in rice, we analyzed the pigment composition of *lyl1-1* using HPLC (Figure 7). In the *lyl1-1* plants, Chl *a* and Chl *b* species are conjugated with incompletely reduced side chains, including Chl_GG_, Chl_DHGG_ and Chl_THGG_, in addition to normal phytylated Chl *a* (Chl *a*_phy_) and Chl *b* (Chl *b*_phy_) (Figure 7A, C, E). A similar change in Chl species was also observed in the *lyl1-2* mutant. However, the *lyl1-2* plants showed preferential accumulation of Chl_GG_, and the amounts of Chl_DHGG_ and Chl_THGG_ species were barely detectable (Figure 7B, D, F). Taken together, these results suggested that the *LYL1* gene participates in the last step of the production of Chl_phy_ molecules in green rice seedlings, and reduced activity of geranylgeranyl reductase leads to the accumulation of Chl intermediates.

**Figure 8:**
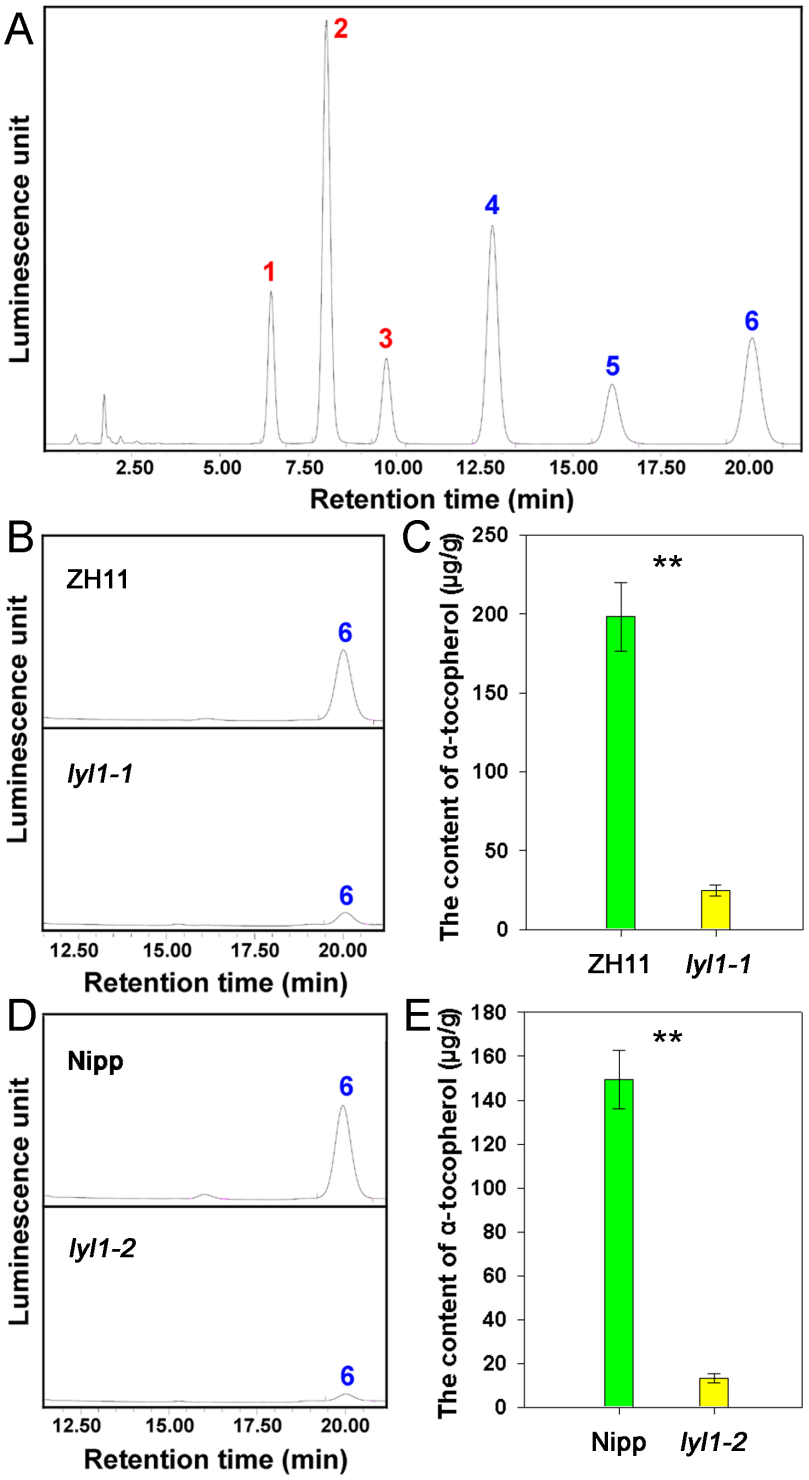
HPLC analysis of tocopherols and tocotrienols. (A) HPLC chromatograms of reference standards. Peak 1, δ-tocotrienol; Peak 2, γ-tocotrienol; Peak 3, α-tocotrienol; Peak 4, δ-tocopherol; Peak 5, γ-tocopherol; Peak 6, α-tocopherol. (B) HPLC chromatograms of extracts from the leaves of ZH11 (top) and *lyl1-1* (bottom), respectively. (C) The contents of α-tocopherol in ZH11 and *lyl1-1* plants. (D) HPLC chromatograms of extracts from the leaves of Nipp (top) and *lyl1-2* (bottom), respectively. (E) The contents of α-tocopherol in Nipp and *lyl1-2* plants. The tocopherols were extracted from the first, second and third leaves of ZH11, *lyl1-1*, Nipp and *lyl1-2* plants grown under natural conditions (high light). Data presented are mean ±SD. ** Significant at the 0.01 level.

PPP is also an obligatory precursor for tocopherol synthesis and is directed into the tocopherol-synthesizing pathway through condensation with homogentisate derived from the shikimate pathway. To further explore the consequences of a mutation in LYL1 protein, we examined the tocopherol content in the *lyl1* mutants (Figure 8). HPLC analysis showed that the α-tocopherol levels in *lyl1-1* and *lyl1-2* were decreased to 12.52% and 8.81% of those in wild type, respectively. This indicated that the reduced activity of geranylgeranyl reductase results in a deficiency of α-tocopherol levels. The *LYL1* gene is also required for the biosynthesis of α-tocopherol in rice.

### *LYL1* protects against lipid peroxidation and ROS

**Figure 9:**
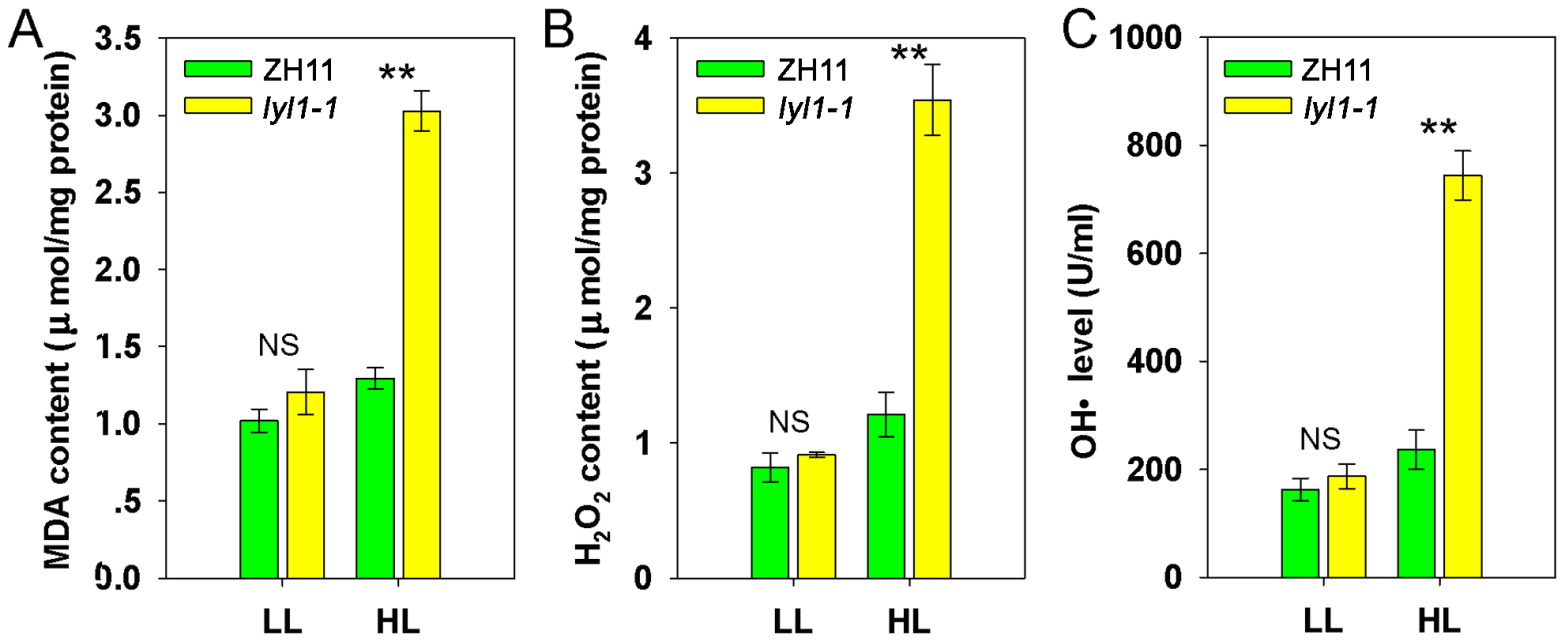
Effects of different light density on the lipid peroxidation and ROS levels. The changes in MDA content (A), H_2_O_2_ content (B) and HO· level (C) between ZH11 and *lyl1-1* plants under low-light (LL, 100 µmol photonm m^−2^ s^−1^) and high-light (HL, 400 µmol photon m^−2^ s^−1^) conditions. Data presented are mean ±SD. NS =  No significant, ** Significant at the 0.01 level.

High-light stress excessively excites lipid peroxidation and generates reactive oxygen species (ROS), which in turn can attack various cellular components. Because the *lyl1* mutants displayed an increased sensitivity to high-light stress, we detected the malondialdehyde (MDA) content, an indicator of lipid peroxidation. Under low-light (100 µmol photon m^−2^ s^−1^) conditions, the content of MDA in the *lyl1-1* and ZH11 plants was similar (Figure 9A). Under high-light (400 µmol photon m^−2^ s^−1^) conditions, the MDA content in the *lyl1-1* leaves was approximate 2.3-fold of that in ZH11 (Figure 9A). We also compared the levels of ROS, including hydrogen peroxide (H_2_O_2_) and hydroxyl radicals (OH·), between ZH11 and *lyl1-1*. As shown in Figure 9, the ROS contents of ZH11 and *lyl1-1* mutant under low-light conditions were indistinguishable. However, ROS levels in the *lyl1-1* mutant were about 2.9-fold and 3.1-fold of those in ZH11 at high-light exposure (Figure 9B, C). These results suggested that the *lyl1-1* mutant senses a higher level of photodamage than wild type and *LYL1* protects plant against lipid peroxidation and ROS.

## Discussion

Chls are essential for photosynthesis. They are responsible for harvesting and transferring solar energy in antenna systems, and for charge separation and electron transport in reaction centers [7]. Chl metabolism is a highly coordinated process that is executed via a series of cooperative reactions catalyzed by numerous enzymes [11]. Analysis of the complete genome of *Arabidopsis* showed that it has 15 enzymes encoded by 27 genes for the biosynthesis of Chl from glutamyl-tRNA to Chl *b* [29]. However, only seven genes encoding five enzymes involved in Chl biosynthesis have been isolated in rice. Jung et al. characterized the *OsCHLH* gene for the OsChlH subunit of magnesium chelatase [30], and Zhang et al. cloned *Chl1* and *Chl9* genes encoding the OsChlD and OsChlI subunits of magnesium chelatase [31]. Chl *b* is synthesized from Chl *a* by Chl *a* oxygenase. Lee et al. and Morita et al. identified *OsCAO1* and *OsCAO2* genes for Chl *a* oxygenase [32], [33]. Chl synthase catalyzes the esterification of chlorophyllide, resulting in the formation of Chl *a*. Wu et al. identified the *YGL1* gene encoding rice Chl synthase [34]. More recently, Wang et al. characterized an 8-vinyl reductase gene responsible for the conversion of divinyl Chl *a* to monovinyl Chl *a* [35].

Although two rice mutants, M249 and M134, that accumulate Chl intermediates with incompletely reduced alcohol side chains were previously characterized [36], the genetic properties of hydrogenating enzyme involved in the final step of Chl biosynthesis in rice are still unknown. In this study, we isolated a mutant, *lyl1-1*, from *japonica* rice c.v. ZH11 treated with ^60^Co. This mutant exhibited dynamic yellow leaves, reduced levels of Chl, arrested development of chloroplasts and a retarded growth rate. Map-based cloning of *LYL1* gene revealed that it encodes a geranylgeranyl reductase.

In the *lyl1-1* mutant, a C-to-T substitution resulted in the change from an alanine residue to valine in the geranylgeranyl reductase. HPLC analysis indicated that the mutant accumulates Chl with incompletely reduced side chains. Under prolonged illumination, the *lyl1-1* plants accumulated intermediates with a similar distribution of side chains in both the Chl *a* and Chl *b* groups in the final step of greening (Figure 7). This suggested that the reduction of side chains occurs in a stepwise manner during the conversion of Chl_GG_ to Chl_phy_ via Chl_DHGG_ and Chl_THGG_ in the synthesis of not only Chl *a* but also Chl *b*. Our results firstly proposed the pathway for the reduction of Chl_GG_ to Chl_phy_ by a hydrogenating enzyme in rice. We also noticed that the *lyl1-1* mutant accumulated all six intermediates and the *lyl1-2* mutant showed preferential accumulation of Chl_GG_. One explanation is that different mutations of *LYL1* affect the preference of hydrogenation of the side chain during complete biosynthesis of Chl_phy_ molecules in green seedlings.

Rice plants require high light to optimize photosynthesis during growth. However, the *lyl1* mutant showed hypersensitivity to high-light stress (Figure 3). Under prolonged illumination, the total Chl content in *lyl1-1* rapidly decreased, whereas the Chl content in ZH11 plants increased. The hypersensitivity of *lyl1-2* was much more severe than that of *lyl1-1*. The *lyl1-2* mutant grew very slowly and died after it was transferred to natural sunlight (data not shown). Direct evidence was further provided by a light-shading experiment (Figure 3). Taken together, our data clearly confirm that the mutation of *LYL1* leads to hypersensitivity to high-light, and the yellowing of leaves in the mutant is caused by light illumination. The increased sensitivity to high-light results from the lack of geranylgeranyl reductase activity is in agreement with previous studies in tobacco [37] and *Synechocystis* sp. PCC 6803 [38].

**Figure 10:**
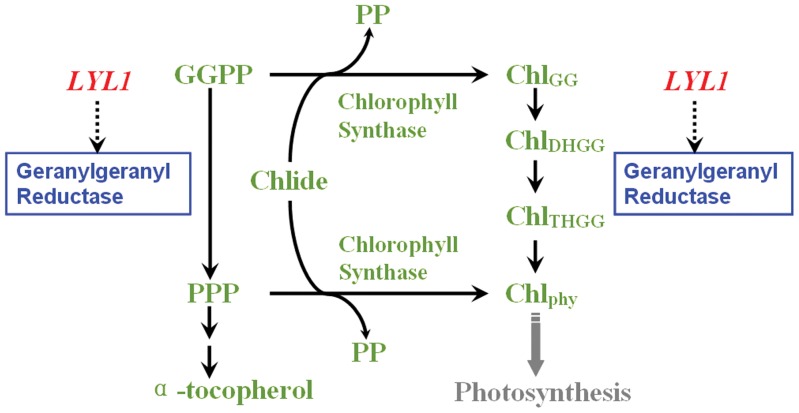
A working model of branched pathway starting from GGPP and Chlide to Chl_phy_ and α-tocopherol in rice. Geranylgeranyl reductase, encoded by the *LYL1*, generates PPP and Chl_phy_ using GGPP and Chlide as substrates. PPP is then directed into the tocopherol-synthesizing pathway.

GGPP is esterified with Chlide to form Chl_GG_, which is subsequently stepwisely reduced to Chl_phy_. Alternatively, GGPP can first be reduced to PPP by geranylgeranyl reductase before it is conjugated with Chlide [18], [19], [25]. PPP forms the hydrophobic carbohydrate side chains of α-tocopherol molecules. The α-tocopherol levels of *lyl1-1* and *lyl1-2* were decreased to 12.52% and 8.81% of those of wild type, respectively (Figure 8). This indicated that *LYL1* gene also contributes to the biosynthesis of α-tocopherol in rice. In addition, the content of α-tocopherol was found to coincide with the total amount of Chl. According to existing literatures and our data presented in this study, we proposed a model that *LYL1* simultaneously participates in the synthesis of Chl and α-tocopherol in rice (Figure 10).

Tocopherol is synthesized by all plants and the common functions are its ability to reduce ROS levels in photosynthetic membranes and to limit the extent of lipid peroxidation by reducing lipid peroxyl radicals to their corresponding hydroperoxides [39], [40]. Tocopherol has long been speculated to have an essential function in protecting photosynthetic organisms against photooxidative stress [41], [42], [43]. However, this long-held assumption in photoprotection was broken by the results from the tocopherol-deficient mutants in *Arabidopsis* and cyanobacteria [44], [45]. The photoautotrophic growth and photoinhibition of the tocopherol-deficient mutants in *Arabidopsis* (*vte* mutants) and *Synechocystis* sp. strain PCC 6803 (slr1736 and slr1737 mutants) was indistinguishable from that of the wild type under high-light stress. Subsequently, a mutant of the cyanobacterium *Synechocystis* sp. PCC 6803 lacking geranylgeranyl reductase, ΔchlP, was compared to strains with specific deficiency in tocopherol to assess the role of Chl *a* phytylatation [46]. The tocopherol-less Δhpt strain growed indistinguishably from the wild type under standard light photoautotrophic conditions, and exhibited only a slightly enhanced rate of photosystem I degradation under strong irradiation. Together with previous data, the results demonstrated that, in the ΔchlP mutant, accumulation of Chl *a*_GG_ instead of deficiency of tocopherol leads to the instability of photosystem. In this study, rice *LYL1* gene mutation reduced the α-tocopherol levels and resulted in yellow plants that had destructive chloroplast membrane system and increased photoinhibition and lipid peroxidation during high-light stress. It is difficult to attribute the observed phenotypes in these experiments directly to reduced α-tocopherol level: mutation of *LYL1* also affects Chl level and causes the accumulation of geranylgeranylated Chl derivatives. Characterization of the mutant with specific deficiency in α-tocopherol will reveal the photoprotective mechanism of *LYL1* in rice.

Recently, it was reported that a mutation in LIL3, one type of LHC-like protein, resulted in the accumulation of Chl molecules conjugated with incompletely reduced side chains and a reduction of α-tocopherol levels in *Arabidopsis* [47]. LIL3 interacts with and stabilizes geranylgeranyl reductase to complete α-tocopherol and Chl biosynthesis. BLAST analysis indicates that there is only one homolog of *LIL3* (LOC_Os02g03330) in the rice genome. This gene, *OsLIL3*, encodes a 250-amino acid protein that has 66% and 58% homology with *LIL3:1* and *LIL3:2*, respectively. We attempted to exam the interaction between the OsLIL3 and LYL1 proteins using the yeast two-hybrid system. However, no significant protein-protein interaction was detected (data not shown). There are several possible explanations for the result. First, the OsLIL3 and LYL1 proteins may not work as a complex in rice. Second, OsLIL3 may function with LYL1 via other proteins. Third, the interaction between OsLIL3 and LYL1 may be too weak to be detected. The molecular nature of the interaction between rice geranylgeranyl reductase and LHC-like proteins should be further studied.

In this study, we characterized a rice light-induced yellow leaf mutant that is hypersensitive to high-light and defective in the Chl_phy_ synthesis. Map-based cloning revealed that *LYL1* encodes a geranylgeranyl reductase. Our data suggest that the *LYL1* gene functions simultaneously in Chl and α-tocopherol synthesis in rice. Our results highlight the critical functions of *LYL1* in the response to light stress and the protection of photooxidative damage in rice.

## Materials and Methods

### Plant materials and growth conditions

The yellow leaf mutant *lyl1-1* was isolated from the progeny of *japonica* rice ZH11 treated with ^60^Co. The mutant was self-pollinated for several generations until the mutation was genetically proven to be truly inherited. Putative Tos17 insertion mutants of the LOC_Os02g51080 gene, *lyl1-2*, were identified by searching the Rice Tos17 Insertion Mutant Database (http://pc7080.abr.affrc.go.jp/~miyao/pub/tos17/index.html.en). Homozygous plants were isolated by PCR screening of seed populations using two sets of primer combinations: D1F, 5′-ATTGTTAGGTTGCAAGTTAGTTAAGA-3′ and D1R, TCATTCACCATTCGTCAGGA; D2F, 5′-TCAAATCAATGGCTGGTCG-3′ and D2R, 5′-ACTTACGCTTGTTCAAATCTGTT-3′ to confirm insertions in the *LYL1* locus.

### Genetic analysis and map-based cloning of *LYL1*

For genetic analysis, an F_2_ population derived from a cross between *lyl1-1* and 9311, an *indica* variety, was grown in paddy fields under natural conditions (high light) and the leaf phenotypes could be clearly identified. This segregating population was also used for locating and fine mapping the *LYL1* locus. Recessive individuals in the F_2_ segregating population were used to screen recombinants. To fine map *LYL1*, InDel markers were developed based on sequence differences between *indica* variety 9311 and *japonica* variety Nipponbare, according to data published in NCBI (http://www.ncbi.nlm.nih.gov). The new polymorphic InDel markers were used to narrow down the region containing *LYL1* (Table S2). Candidate genes were amplified and sequenced using gene-specific primers. A single nucleotide substitution of the putative *lyl1-1* allele in the mutant was detected with a Cleaved Amplified Polymorphic Sequences (CAPS) marker using the primer pair 5′-GAGGAGAAGCCACAGAAACG-3′ and 5′-TCTTGGTGAGGCAGTAGTAATAAA-3′, followed by digestion with *Sac*II. The sequence of *LYL1* and *lyl1-1* has been deposited into the NCBI/GenBank with an accession number KF305678 and KF305679.

For RNAi analysis, a DNA fragment of LOC_Os02g51080 was amplified by PCR using the primer pair 5′-AAAGGATCCCCGCTGTGCATGGTGTC-3′ and 5′-AAAACTAGTATGTCGGGCTTGTGGGT-3′. This fragment was cloned into the pMD18-T vector (TaKaRa) and sequentially cloned into the *BamH*I/*Spe*I and *Bgl*II/*Xba*I sites of the p1022 vector. Then, the stem-loop fragment was cloned into the p1301UbiNOS vector [48]. The resulting RNAi construct was transformed into *A. tumefaciens* and used for further transformation.

### RNA extraction and quantitative real-time PCR

Total RNA was extracted from various tissues of ZH11 and *lyl1-1* plants using Trizol reagent (Invitrogen) and treated with *DNase I* (TaKaRa) following the manufacturer's protocol. Approximately 1 µg of total RNA from each sample was used for first-strand cDNA synthesis. For quantitative real-time RT-PCR, first strand cDNAs were used as templates in reactions using SYBR Green PCR Master Mix (Takara) according to the manufacturer's instructions. *OsActin* gene was amplified as a control. Amplification of target genes was carried out using an ABI 7500 Real-time System. PCR was performed with the following primer sets: *LYL1*, 5′-GCGGATGGTGGAGGAGA-3′ and 5′-TGCCGATGGTGTTGACG-3′; *OsActin*: 5′-GATGACCCAGATCATGTTTG-3′ and 5′-GGGCGATGTAGGAAAGC-3′.

### Transmission electron microscopy

The leaf samples of ZH11 and *lyl1-1* plants were harvested from 1-month-old plants grown under natural conditions (high light). Leaf sections were fixed in 2% glutaraldehyde and further fixed in 1% OsO_4_. Tissues were stained with uranyl acetate, dehydrated in ethanol and embedded in Spurr's medium prior to thin sectioning. Samples were stained again and examined with a HITACHI H-600 transmission electron microscope.

### Analysis of pigments and α-tocopherol

Chls were extracted from 0.2 g fresh leaves of ZH11 and the mutant with 95% ethanol, and Chl contents were determined with a spectrophotometer according to the previous method [34]. For analysis of Chl intermediates, approximately 5 mg of tissue from the leaves of light-grown seedlings or greening coleoptiles of dark-grown seedlings were weighed and processed in a chilled homogenizer with aqueous acetone, as previously described [36], [47]. The homogenates were clarified by centrifugation. The extracts (10∼20 µl) were injected onto an ODS-C18 reverse-phase column (Agilent, 4.6×250 mm length, 2.5 µm) and eluted at 40°C with 100% methanol at a flow rate of 1.5 ml/min. The fluorescence intensity at 650 nm of chlorophyll in the eluate excited at 440 nm was monitored with a fluorescence detector. The amount of Chl *a* and *b* intermediates was calculated according to a previous method [36]. Three independent biological repeats were performed.

Tocopherol was extracted using previous methods [49], [50]; approximately 0.3 grams of rice leaves was saponified under nitrogen in a screw-capped tube with 2 ml of potassium hydroxide (600 g/l), 10 ml of ethanol, 2 ml of sodium chloride (10 g/l) and 5 ml of ethanolic pyrogallol (60 g/l) added as an antioxidant. Tocopherol was determined using an Agilent 1200 HPLC. Resolution of vitamin E species was achieved using an Agilent Eclipse XDB-C18 column (4.6×150 mm length, 5 µm) and a solvent system consisting of methanol: water (95∶5, v/v) with a flow rate of 1.5 ml/min. Sample components were detected and quantified by fluorescence with excitation at 292 nm and emission at 330 nm. A sample volume of 10 µl was injected for chromatographic analysis. Three independent biological repeats were performed.

### Western-blot analysis

The thylakoid membrane proteins of leaves were extracted according to a previous method [51]. And the protein content was determined by spectrophotometer using bovine serum albumin (BSA) standard as a reference. About 20 mg proteins were mixed with 5X loading buffer (250 mM Tris-HCl, pH 6.8, 50% glycerol, 10% SDS, 5% 2-mercaptoetnanol, 0.5% bromophenol blue). This mixture was boiled for 5 minutes and loaded onto a 12% SDS-PAGE gel. The proteins were separated and transferred onto a Nitrocellulose Transfer Membrane (Whatman) by electrophoretic cell (Bio-rad) with transfer buffer (25 mM Tris-base, 192 mM glycine, 20% methanol, pH 8.3). The membranes were blocked with Tris-HCl buffer containing 0.15 M NaCl and 5% non-fat dry milk, and were probed with primary antibodies (at a dilution of 1∶200 in PBS) specific for LHC I subunits (Lhca1, Lhca2, Lhca3, and Lhca4) and LHC II subunits (Lhcb1, Lhcb2, Lhcb4, and Lhcb5), which were purchased from Agrisera. Then membranes were incubated with the alkaline phosphatase-conjugated secondary antibody (Sigma) (1∶2000). The final substrates (NBT and BCIP) were added for color development [52], [53].

### ROS scavenging and lipid peroxidation determination

The level of lipid peroxidation was estimated in term of MDA content determined by thiobarbituric acid (TBA) reaction. About 200 mg tissue was homogenized with 5 ml 0.25% TBA. The homogenate was boiled for 40 min at 95°C and centrifuged at 13,000 g for 10 min. The absorbance of the supernatant was recorded at 532 nm and corrected by substracting absorbance at 600 nm. For the estimation of H_2_O_2_, about 200 mg tissue was homogenized in 0.9% physiological saline in a chilled pestle and mortar. The homogenate was centrifuged at 10,000 g for 10 min at 4°C and the supernatant was used for the detection of H_2_O_2_. The HO· level was determined spectrophotometrically based on the increase in absorbance of H_2_O_2_ at 550 nm. In this assay, one unit of HO· is defined as the capability increasing the accumulation of 1 mmol of H_2_O_2_ per milliliter. The HO· level was expressed as unit/mg protein. Protein content was determined according to the method of Bradford using bovine serum albumin as standard [54].

All the experiments were carried out with the kits (Nanjing Jiancheng Bioengineering Institute) according to the manufacturer's instructions. Three independent biological repeats were performed.

### Phylogenetic trees analysis

To identify green plant genes encoding geranylgeranyl reductase, BLASTP searches were performed in the Phytozome database with the amino acid sequence of the rice gene *LYL1* used as a query. If a protein sequence satisfied *E*≤10^−10^, it was selected as a candidate protein.

To identify the geranylgeranyl reductase in eukaryotic genome, BLAST searches against the non-redundant (*nr*) protein sequence database, NCBI EST database and available eukaryotic genome databases were performed using plant geranylgeranyl reductase sequences as queries. Protein sequences were sampled for further combined phylogenetic analysis from representative groups within each domain of life (bacteria, archaea and eukaryotes) based on BLASTP results against the *nr* database.

All of the selected representative protein sequences were aligned using Clustal X [55]. The gaps and ambiguously aligned sites were removed manually. Phylogenetic analysis was performed with a maximum likelihood approach using PhyML version 3.0 [56] and a Neighbor-joining method using MEGA [57]. A total of 100 non-parametric bootstrap samplings were performed to estimate the support level for each internal branch. Phylogenetic trees were visualized using the *Explorer* program of MEGA.

## Supporting Information

Figure S1 Phylogenetic analysis of the LYL1 homologs. The numbers above the branches show bootstrap values for maximum likelihood and distance analysis, respectively. Asterisks indicate values lower than 50%.
(TIF)

Figure S2 Amino acid sequence alignment of LIL1 and other geranylgeranyl reductase proteins. Residues conserved across three or more sequences are shaded black, and similar residues conserved across three or more sequences are shaded gray. Numbers correspond to amino acid positions.
(TIF)

Table S1 List of geranylgeranyl reductase genes in 14 representative plants.
(DOC)

Table S2 Primers used for fine mapping in this study.
(DOC)
