# Supplementary figures and images for: Mutation of the Light-Induced Yellow Leaf 1 Gene, Which Encodes a Geranylgeranyl Reductase, Affects Chlorophyll Biosynthesis and Light Sensitivity in Rice
Source: PLoS One. 2013 Sep 10;8(9):e75299. doi: 10.1371/journal.pone.0075299 (PMC3769248; doi:10.1371/journal.pone.0075299)

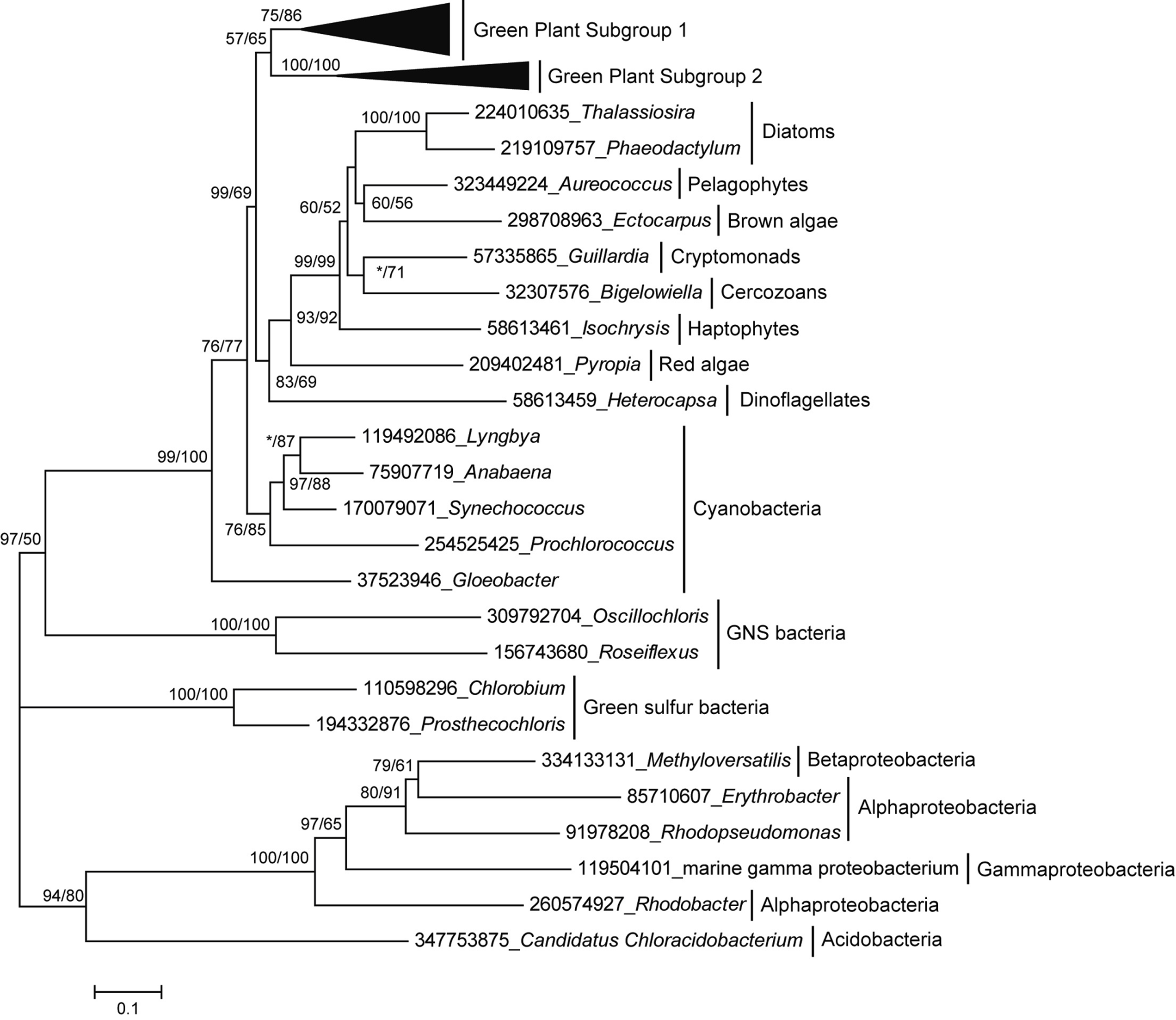

Supplement: Figure S1 — Phylogenetic analysis of the LYL1 homologs. The numbers above the branches show bootstrap values for maximum likelihood and distance analysis, respectively. Asterisks indicate values lower than 50%. (TIF) [file pone.0075299.s001.tif]

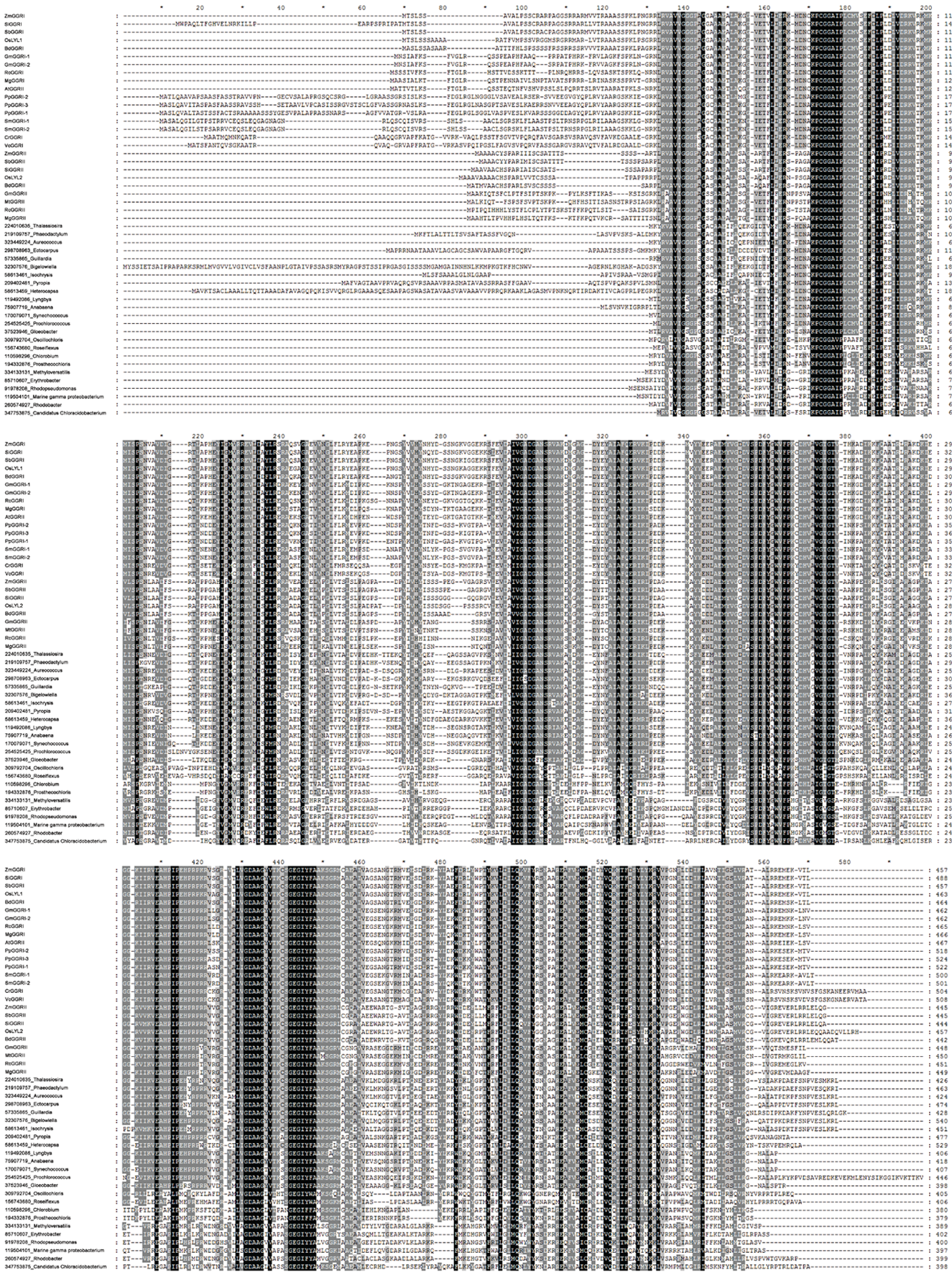

Supplement: Figure S2 — Amino acid sequence alignment of LIL1 and other geranylgeranyl reductase proteins. Residues conserved across three or more sequences are shaded black, and similar residues conserved across three or more sequences are shaded gray. Numbers correspond to amino acid positions. (TIF) [file pone.0075299.s002.tif]
