# Supplementary material for: Mutation of the Light-Induced Yellow Leaf 1 Gene, Which Encodes a Geranylgeranyl Reductase, Affects Chlorophyll Biosynthesis and Light Sensitivity in Rice
Source: PLoS One. 2013 Sep 10;8(9):e75299. doi: 10.1371/journal.pone.0075299 (PMC3769248; doi:10.1371/journal.pone.0075299)
Supplement: Table S1 — List of geranylgeranyl reductase genes in 14 representative plants. (DOC) [file pone.0075299.s003.doc]

**Table S1 List of geranylgeranyl reductase** genes in 14 representative plants.

| Lineage | Species | Gene | Locus | Length | Intron | Chr/scaffold | Location |
| --- | --- | --- | --- | --- | --- | --- | --- |
| Monocots | *Oryza sativa* | *OsLYL2* | Os01g16020 | 457 | 0 | Chr 1 | 9018144 - 9019762 |
|  | *Oryza sativa* | *OsLYL1* | LYL1 | 463 | 2 | Chr 2 | 31246225 - 31248818 |
|  | *Zea mays* | *ZmGGRII* | Zm2g419111 | 445 | 0 | Chr 3 | 40062008 - 40064270 |
|  | *Zea mays* | *ZmGGRI* | Zm2g105644 | 457 | 2 | Chr 5 | 206890298 - 206892838 |
|  | *Setaria italica* | *SiGGRI* | Si017037m | 488 | 2 | scaffold_1 | 37904962 - 37908092 |
|  | *Setaria italica* | *SiGGRII* | Si004482m | 444 | 0 | scaffold_5 | 4622979 - 4624313 |
|  | *Sorghum bicolor* | *SbGGRII* | Sb03g010340 | 445 | 0 | Chr 3 | 11199021 - 11200358 |
|  | *Sorghum bicolor* | *SbGGRI* | Sb04g028050 | 457 | 2 | Chr 4 | 57992921 - 57995090 |
|  | *Brachypodium distachyon* | *BdGGRII* | Bd2g09777 | 442 | 0 | Bd2 | 8021252 - 8022580 |
|  | *Brachypodium distachyon* | *BdGGRI* | Bd3g59430 | 464 | 2 | Bd3 | 58604547 - 58606805 |
| Eudicots | *Arabidopsis thaliana* | *AtGGRII* | At1g74470 | 467 | 2 | Chr 1 | 27991165 - 27993006 |
|  | *Glycine max* | *GmGGRII* | Gm02g44150 | 448 | 0 | Chr 2 | 48789243 - 48790727 |
|  | *Glycine max* | *GmGGRI-1* | Gm05g01000 | 462 | 1 | Chr 5 | 606608 - 608812 |
|  | *Glycine max* | *GmGGRI-2* | Gm17g10890 | 462 | 1 | Chr 17 | 8191012 - 8193974 |
|  | *Medicago truncatula* | *MtGGRII* | Mt5g094790 | 450 | 0 | Chr 5 | 37548583 - 37550059 |
|  | *Ricinus communis* | *RcGGRII* | Rc29942.t000018 | 451 | 0 | 29942 | 275682 - 277579 |
|  | *Ricinus communis* | *RcGGRI* | Rc30138.t000242 | 465 | 1 | 30138 | 1654120 - 1655862 |
|  | *Mimulus guttatus* | *MgGGRI* | Mg1a027074m | 466 | 0 | scaffold_20 | 1068543 - 1069943 |
|  | *Mimulus guttatus* | *MgGGRII* | Mg1a026544m | 449 | 0 | scaffold_173 | 7571 - 8920 |
| Mosses | *Physcomitrella patens* | *PpGGRI-1* | Pp1s20_284V6 | 522 | 0 | scaffold_20 | 1933511 - 1935446 |
|  | *Physcomitrella patens* | *PpGGRI-2* | Pp1s100_107V6 | 518 | 0 | scaffold_100 | 529068 - 531241 |
|  | *Physcomitrella patens* | *PpGGRI-3* | Pp1s425_20V6 | 524 | 0 | scaffold_425 | 170238 - 172454 |
| Lycophyte | *Selaginella moellendorffii* | *SmGGRI-2* | Sm150713 | 502 | 0 | scaffold_28 | 1313504-1315105 |
|  | *Selaginella moellendorffii* | *SmGGRI-1* | Sm271030 | 501 | 0 | scaffold_22 | 1012506-1014092 |
| Green algae | *Chlamydomonas reinhardtii* | *CrGGRI* | Cr01g050950 | 504 | 4 | Chr 1 | 7001865 - 7005687 |
|  | *Volvox carteri* | *VcGGRI* | Vc20009211m | 508 | 4 | scaffold_1 | 4718509 - 4722451 |
