# Supplementary material for: Mutation of the Light-Induced Yellow Leaf 1 Gene, Which Encodes a Geranylgeranyl Reductase, Affects Chlorophyll Biosynthesis and Light Sensitivity in Rice
Source: PLoS One. 2013 Sep 10;8(9):e75299. doi: 10.1371/journal.pone.0075299 (PMC3769248; doi:10.1371/journal.pone.0075299)
Supplement: Table S2 — Primers used for fine mapping in this study. (DOC) [file pone.0075299.s004.doc]

**Table S2 Primers used for fine mapping in this study.**

| Markers | Forward sequence (5′-3′) | Reverse sequence (5′-3′) |
| --- | --- | --- |
| W226 | GATGGTGGGACTGGCTAGTGT | TGTGGTGGTTATGTTGGGAAG |
| W232 | CTGCCTCACTGTTCATCC | TACGTCGTCATTAAGGTTGT |
| W235 | TCTCACCGTCATATTCCA | CTCCACTCTACTTCCACCT |
| W243 | CTTAACATCAACACCGATTC | TCCCAACTCAAACTACCG |
| W246 | ATTTGTCGGTGTCGTTGA | GGGGACTGAGGGGTAATA |
| W247 | GCCCAAGTCATTCAACAA | CTAGGCTACATGCCCATT |
| W248 | GCCTCTACATTTCACTTACCA | CCCAAGGTTAATAGTTTCG |
| W251 | CATAAATCGGTTTGGAGA | GCGTATTAGCGGAACAT |
| W252 | ATTCTCCGATACTATACTTTGC | TCCCAATGATTTACAGGTT |
| W256 | CATGCAGTATGAGGGACG | ATTAGACACGCAGTTACGC |
